# Consumer Health Information Services and Programs: Best Practices

**DOI:** 10.5195/jmla.2017.118

**Published:** 2017-01

**Authors:** Carolyn G. Biglow

One of the most important aspects of consumer health information services and programs is the emphasis on best practices. As a consumer health librarian, I am always looking for successful examples of services and programs that I can offer to the patients and families of my hospital. This book is an invaluable resource for consumer health librarians, especially those who are solo, as I am. I usually have to attend the Medical Library Association (MLA) annual meeting or solicit ideas from MEDLIB-L readers to find out about ideas that work.

M. Sandra Wood, FMLA, has brought together a wide range of librarians who have experience in all types of consumer health libraries. In the first chapter by Cara Marcus, AHIP, of Brigham and Women’s Faulkner Hospital Patient/Family Resource Center, I found many ideas that would benefit the patients and families who visit my family resource center, from proactively selecting materials that focus on hospital services to providing finding aids for users who are not familiar with library or medical terminology.

Also of great interest is the chapter by Jacqueline M. Davis, the consumer health librarian at Sharp HealthCare’s Consumer Health Library. This chapter deals with a subject that should particularly interest all consumer health information librarians: health literacy and its role in supporting patients and families as equal partners in their own and their families’ health care. Davis has detailed her library’s determination to provide her patrons with the correct amount of relevant, understandable, and reliable medical information that they want and need.

Whether you are looking for ideas while planning your consumer health information library service or are trying to find successful enhancements for your existing program, you will find many interesting and patron-oriented best practices in this book. It is very well written and contains detailed information that will apply to a wide range of consumer health libraries. Up-to-date resources are also included, as well as information on the backgrounds and experience of each chapter’s author. Any consumer health information librarian would be wise to include this book in his or her collection of essential resources.

